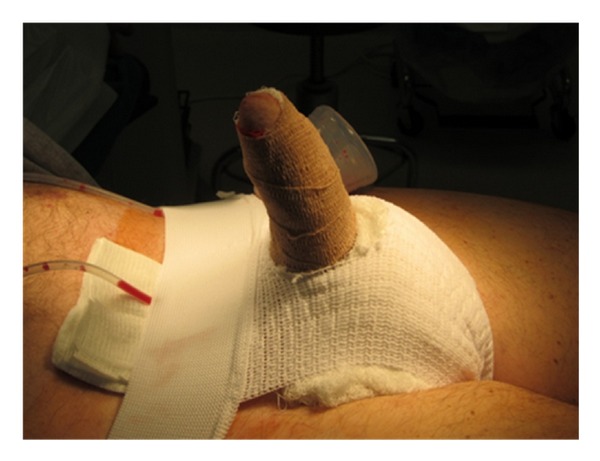# Streamlined Approach for Infrapubic Placement of an Inflatable Penile Prosthesis

**DOI:** 10.1155/2012/520180

**Published:** 2012-05-28

**Authors:** Edward Karpman

**Affiliations:** El Camino Urology Medical Group, Mountain View, CA 94040, USA

## Abstract

The streamlined approach for infrapubic placement of an inflatable penile prosthesis is a variation of the traditional infrapubic approach. A better understanding of operative techniques and recent clinical outcome studies have led to an evolution of the original infrapubic approach. Small incisions and efficient operative maneuvers can shorten operative times and expedite postoperative recovery.

## 1. Introduction

The penile prosthesis is the gold standard of treatment for men with erectile dysfunction (ED) refractory to more conservative therapy [1]. Placement of the inflatable penile prosthesis (IPP) has been reported from several different approaches including penoscrotal, infrapubic, suprapubic, and perineal locations [2–4]. Each approach has its own unique advantages and disadvantages and it is ideal that any surgeon performing penile prosthetic surgery has some familiarity with at least a few of the approaches. Oftentimes, unique patient anatomy or previous surgery can make one approach more difficult, or easier, over another. The penoscrotal (PS) technique is currently the most popular approach with approximately 80% of IPPs placed in this manner based on manufacturer data [5].

 Historically, the suprapubic approach was the initial approach for IPP placement [2]. Large incisions were required to bury the tubing under the fascia to prevent kinking and malfunction. The development of kink-resistant tubing was one of the major advancements for IPP surgery allowing for smaller incisions and more options for surgical incision sites. The infrapubic approach (IP) was a natural transition to a less invasive approach utilizing the same principles and orientation of the suprapubic approach. The penoscrotal approach was developed shortly thereafter. It offered the advantages of close proximity to the corpora, visualization for placement, and fixation of the pump in the scrotum and less concern for damaging the penile neurovascular bundle. However, the PS approach made reservoir placement more difficult raising the potential risk of reservoir herniation postoperatively [6].

 The streamlined approach for infrapubic placement of a penile prosthesis is a variation of the traditional IP approach. This approach utilizes smaller incisions and corporotomies, minimizes corporal dilation and decreases tissue dissection allowing for shorter operative times and early return to sexual function.

## 2. Technique

### 2.1. Patient Preparation

The patient is laid supine on the operating table with the table flexed. The bladder is catheterized and the catheter is removed. The patient is prepped with 70% isopropyl alcohol, Hibiclens, and ChloraPrep [7, 8]. An artificial erection is established with rapid infusion through an 18 gauge butterfly needle of dilute lidocaine and saline solution prior to incision (10 mL 1% lidocaine and 50 mL saline) after manual occlusion of the base of the penis. This helps identify any penile deformity such as Peyronie's disease, hydrodistend the corpora, and establishing local anesthesia in the penis.

### 2.2. Incision

**Figure 1:**
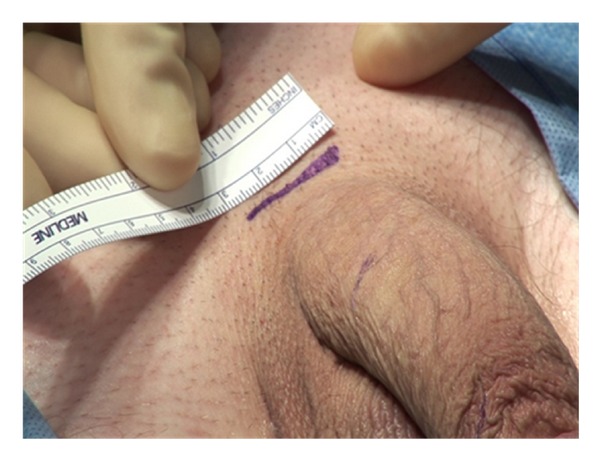


A 3 cm incision is made at the inferior border of the pubis (Figure 1). In obese patients, the incision should be made closer to the penis to facilitate identification and dilation of the corpora. Sharp dissection is carried down through Scarpa's fascia.

**Figure 2:**
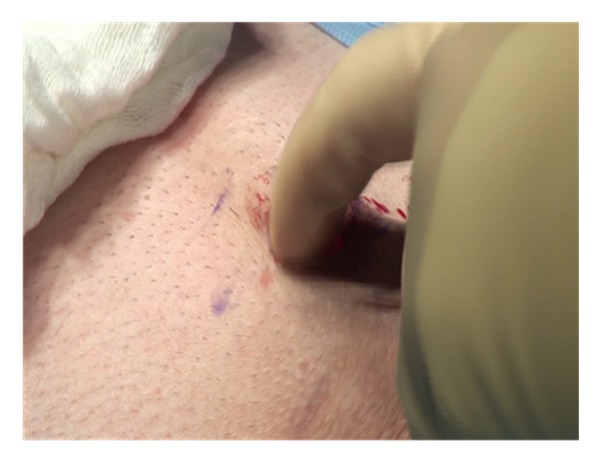


Blunt manual dissection is then used to develop a space down to the corpora and on each side of the corpora (Figure 2).

**Figure 3:**
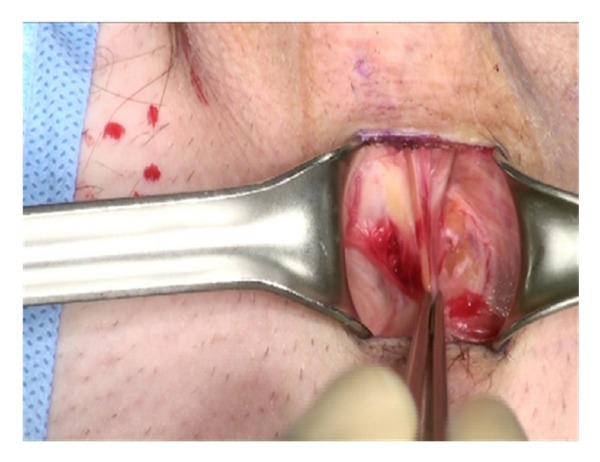


Lone Star retractors are not necessary for the IP approach. Two-hand held retractors give adequate exposure. The corpora are easily visualized and the neurovascular bundle is identified and avoided during the corporotomies (Figure 3).

**Figure 4:**
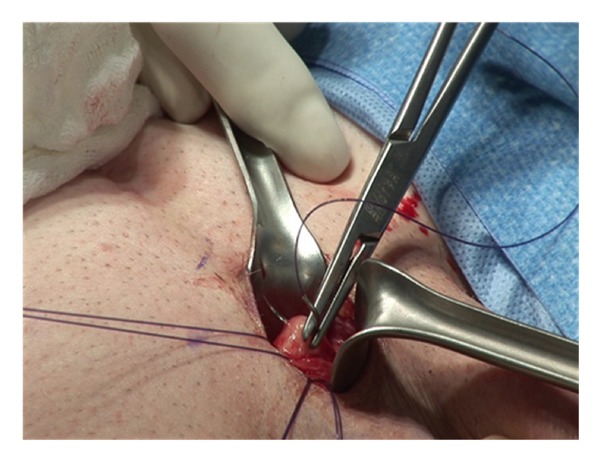


Two 2-0 Monofilament stay sutures are placed in a parallel fashion in each corpora (Figure 4).

**Figure 5:**
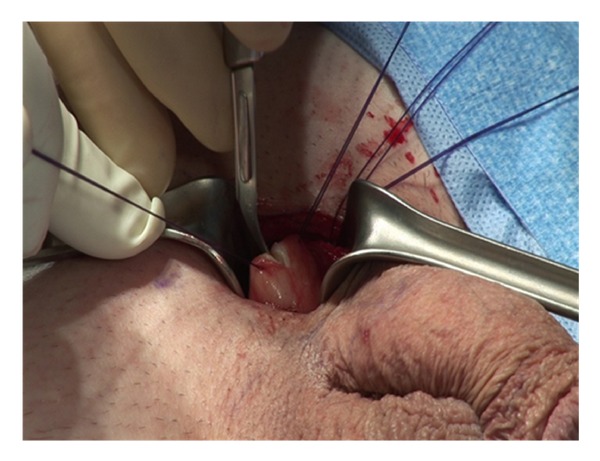


A corporotomy is made in between the stay sutures using a number 12 scalpel (Figure 5). The length of the corporotomy is 1.5 cm, the minimum size to accommodate the proximal end of the penile implant.

**Figure 6:**
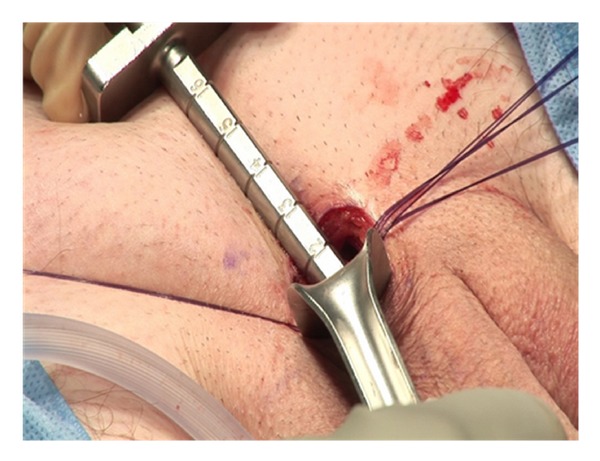


The Furlow instrument is passed once proximally and once distally with simultaneous measurement (Figure 6). Sequential dilation is not required during most first time penile implant surgeries unless there is a history of priapism [9]. A distal fluid challenge can be performed after dilation to exclude the possibility of a distal urethral perforation. This is accomplished by irrigating the distal corpora with a bulb syringe. Fluid exiting from the urethra confirms a distal perforation. Additionally, a field goal test is performed to exclude proximal urethral perforation by placing two Brooks dilators simultaneously in the proximal corpora. The level appearance of the dilators in the form of a “field goal” excludes a proximal perforation.

**Figure 7:**
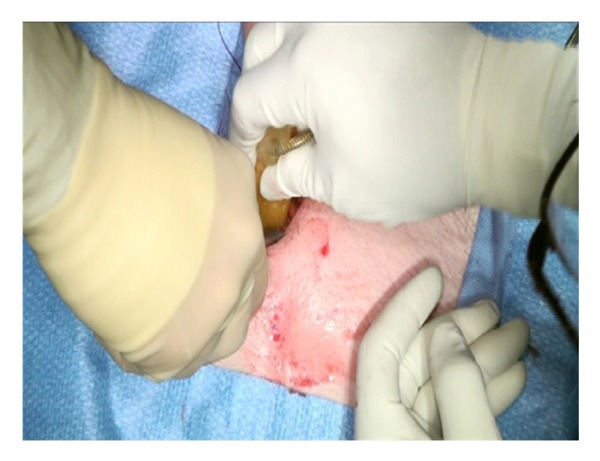


The reservoir is placed through the external inguinal ring with the aid of a 4 inch nasal speculum (Figure 7). The external inguinal ring is easily visualized through the IP incision. Both traditional placements in the space of Retzius and ectopic placement are facilitated by the IP approach. The bladder should be decompressed prior to placement of the reservoir to prevent iatrogenic bladder injury.

**Figure 8:**
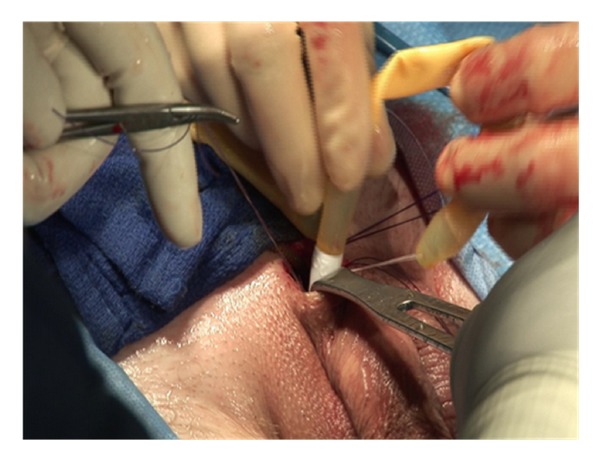


After measuring the corpora, the appropriate implant is selected and prepared for implantation (Figure 8). The Furlow instrument is used to seat the implant proximally and distally.

**Figure 9:**
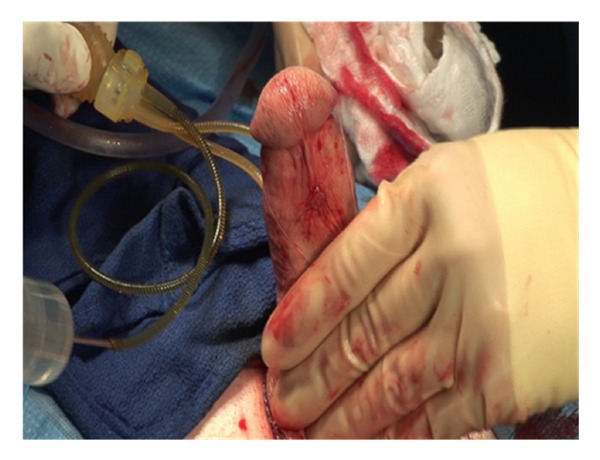


The implant is inflated prior to closure of the corporotomy, further seating the implant proximally and distally. The tips of the implant should be confirmed to be in the mid glans of the penis, the cylinders in each corpora and no buckling or folding of the implant (Figure 9). If there is any concern for crossover, the implant should be removed and redilated with a Hagar dilator in the presumably normal corpora.

**Figure 10:**
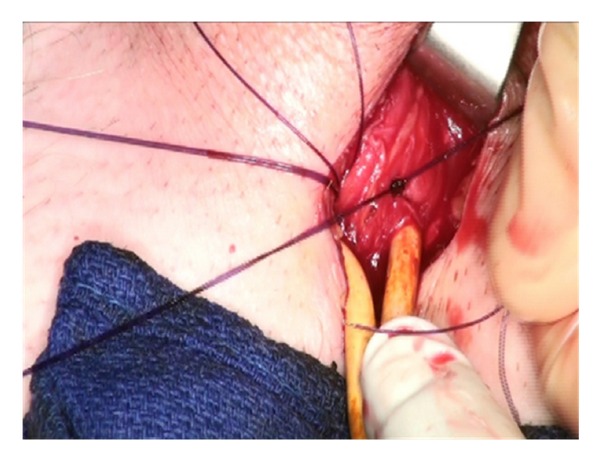


The corporotomies are closed with the 2 previously placed stay sutures. Additional suture placement is not required for watertight closure if the corporotomies are kept to a minimum (Figure 10).

**Figure 11:**
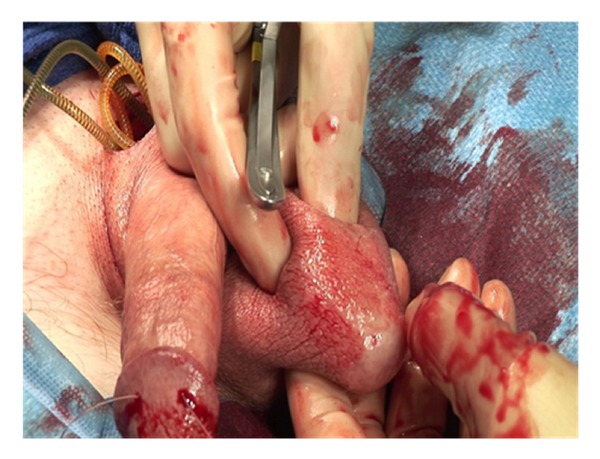


The pump is then placed in the scrotum (Figure 11). A 4-inch nasal speculum is passed lateral to the corpora and posterior and medial to the testes creating a space. The pump is easily placed in the pocket created by the open jaws of the nasal speculum. It is important to note that this is the extent of scrotal surgery and dissection during the IP approach.

**Figure 12:**
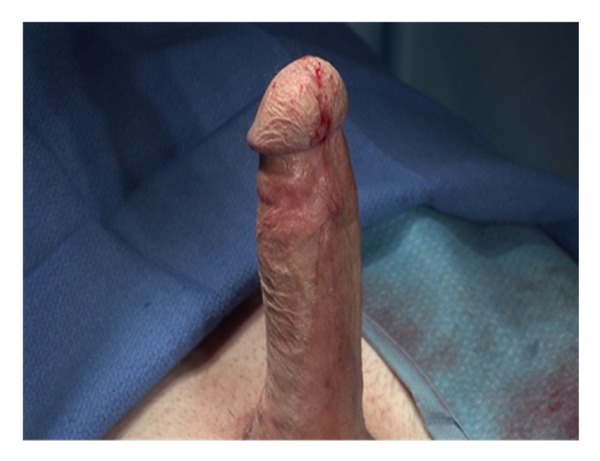


The redundant tubing is trimmed and the manufacturer-provided quick connector system is used to connect the pump to the reservoir. A closed suction drain is left in the dependent portion of the ipsilateral hemiscrotum of the pump. The implant is left fully inflated (Figure 12).

The penis and scrotum are wrapped in a modified Mummy dressing and scrotal support [10] (Figure 13).

## 3. Conclusion

 Prosthetic surgeons should be familiar with more than one approach for placement of an inflatable penile prosthesis. The streamlined infrapubic approach is a variation of the traditional IP approach and may benefit the surgeon in terms of efficiency and the patient in terms of recovery.

## Figures and Tables

**Figure 13:**