# Erratum to “Streamlined Approach for Infrapubic Placement of an Inflatable Penile Prosthesis”

**DOI:** 10.1155/2012/984809

**Published:** 2012-09-04

**Authors:** Edward Karpman

**Affiliations:** El Camino Urology Medical Group, Mountain View, CA 94040, USA

With reference to the paper “Streamlined Approach for Infrapubic Placement of an Inflatable Penile Prosthesis”, we want to change in the introduction section paragraph 3 on page 1 to read “our streamlined approach for infrapubic placement of penile prosthesis is a variation of the minimally invasive approach originally published by Perito [1, 2].” 
